# Synthesis and Characterization of Some New *Bis*-Pyrazolyl-Thiazoles Incorporating the Thiophene Moiety as Potent Anti-Tumor Agents

**DOI:** 10.3390/ijms17091499

**Published:** 2016-09-07

**Authors:** Sobhi M. Gomha, Mastoura M. Edrees, Farag M. A. Altalbawy

**Affiliations:** 1 Department of Chemistry, Faculty of Science, Cairo University, Giza 12613, Egypt; s.m.gomha@gmail.com; 2 Department of Organic Chemistry, National Organization for Drug Control and Research (NODCAR), Giza 12311, Egypt; mmohamededrees@yahoo.com; 3 Faculty of Science, King Khalid University, Abha 61413, Saudi Arabia; 4 Department of Measurements and Environmental Applications, National Institute of Laser Enhanced Sciences (NILES), Cairo University, Giza 12613, Egypt

**Keywords:** *bis*-heterocycles, *bis*-chalcones, hydrazonoyl halides, anticancer agents

## Abstract

A new series of 1,4-*bis*(1-(5-(aryldiazenyl)thiazol-2-yl)-5-(thiophen-2-yl)-4,5-dihydro-1*H*-pyrazol-3-yl)benzenes **3a**–**i** were synthesized via reaction of 5,5′-(1,4-phenylene)*bis*(3-(thiophen-2-yl)-4,5-dihydro-1*H*-pyrazole-1-carbothioamide) (**1**) with hydrazonoyl halides **2a**–**i**. In addition, reaction of **1** with ethyl chloroacetate afforded *bis*-thiazolone derivative **8** as the end product. Reaction of compound **8** with methyl glyoxalate gave *bis*-thiazolone derivative **10**. The structures of the newly synthesized compounds were established on the basis of spectroscopic evidences and their alternative syntheses. All the synthesized compounds were evaluated for their anti-tumor activities against hepatocellular carcinoma (HepG2) cell lines, and the results revealed promising activities of compounds **3g**, **5e**, **3e**, **10**, **5f**, **3i**, and **3f** with IC_50_ equal 1.37 ± 0.15, 1.41 ± 0.17, 1.62 ± 0.20, 1.86 ± 0.20, 1.93 ± 0.08, 2.03 ± 0.25, and 2.09 ± 0.19 μM, respectively.

## 1. Introduction

Thiophene and their *bis*-heterocycles have received considerable attention during the last two decades, as they are endowed with a wide range of therapeutic properties, such as analgesic, antibacterial, antioxidant, anti-inflammatory, antifungal, anticancer, and local anesthetic activities [1,2,3,4,5,6]. On the other hand, pyrazolines have been reported to possess a variety of significant and diverse pharmacological activities such as antibacterial [7], antifungal [8], anticonvulsant [9], DPPH radical scavenging and anti-diabetic [10], antitubercular [11], antidepressant [12], anti-inflammatory [13], antiamebic [14], analgesic [15], and anticancer [16] activities. Moreover, thiazoles are a familiar group of heterocyclic compounds possessing a wide variety of biological activities such as antimicrobial [17], antioxidant [18], antitubercular [19], anticonvulsant [20], anticancer [21,22,23,24,25,26,27], and anti-inflammatory [28] agents.

In view of these reports and in continuation of our previous works in synthesis of bioactive *bis*-heterocyclic compounds [29,30,31], we are herein interested in synthesis of *bis*-pyrazolyl-thiazoles incorporating the thiophene moiety using the hitherto unreported 5,5′-(1,4-phenylene)*bis*(3-(thiophen-2-yl)-4,5-dihydro-1*H*-pyrazole-1-carbothioamide) as versatile building blocks for the synthesis of the title compounds. All the newly synthesized products were screened for their anti-tumor activities against hepatocellular carcinoma (HepG2) cell lines and showed activities with good IC_50_ for seven compounds.

## 2. Results

### 2.1. Synthesis

**Scheme 1:**
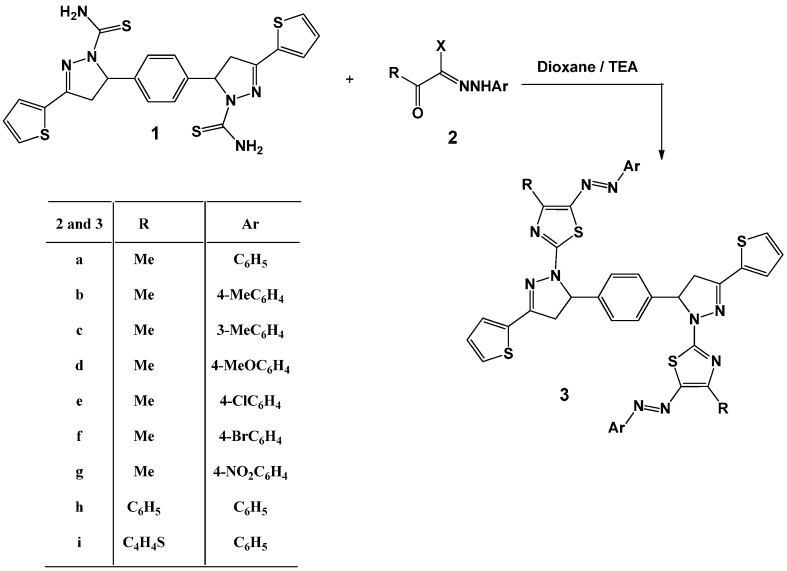
Synthesis of *bis*-arylazothiazole derivatives **3a**–**i**.

The reactivity of 5,5′-(1,4-phenylene)*bis*(3-(thiophen-2-yl)-4,5-dihydro-1*H*-pyrazole-1-carbothio-amide) (**1**) [32] was investigated towards hydrazonoyl halides aiming to synthesize new *bis*-heterocyclic compounds containing 1,3-thiazole ring. Thus, refluxing compound **1** with two moles of each of the hydrazonoyl halides **2a**–**i** in dioxane in the presence of triethylamine gave, in each case, a single product, as indicated by TLC analysis of the crude product, with 68%–76% yield. The structure of the product was identified as 1,4-*bis*(1-(5-(aryldiazenyl)thiazol-2-yl)-3-(thiophen-2-yl)-4,5-dihydro-1*H*-pyrazol-5-yl)benzene **3** as outlined in Scheme 1 based on its elemental analysis and spectral (IR, ^1^H NMR and MS) data [21,24,33]. For example, the ^1^H NMR spectra of compound **3a**, taken as a typical example of the series, showed six signals at δ 2.59 (s, 6H, 2CH_3_), 3.15–3.19 (m, 2H, 2CH-pyrazoline), 3.77–3.82 (m, 2H, 2CH-pyrazoline), 5.92–5.96 (m, 2H, 2CH-pyrazoline), 7.02–7.82 (m, 16H, Ar-H and thiophene-H), and 7.99 (s, 4H, Ar-H). The mass spectrum of **3a** showed a peak corresponding to its molecular ion at *m*/*z* 780 (see Material and Methods).

**Scheme 2:**
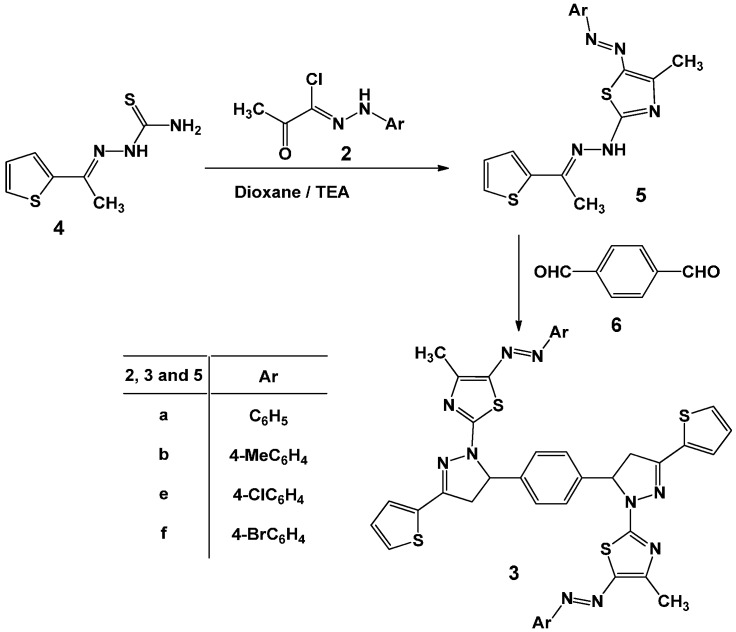
Alternate synthesis of *bis*-arylazothiazole derivatives **3a**,**b**,**e**,**f**.

Next, the reaction of 2-(1-(thiophen-2-yl)ethylidene)hydrazinecarbothioamide (**4**) [34] with 2-oxo-*N*′-arypropanehydrazonoyl chlorides **2a**,**b**,**e**,**f** in dioxane under reflux in the presence of TEA as a basic catalyst afforded one isolable product (as evidenced by TLC analysis of the crude product), which were identified to be arylazothiazole derivatives **5a**,**b**,**e**,**f** (72%–76% yield) as outlined in Scheme 2. The structure of compounds **5a**,**b**,**e**,**f** was elucidated by elemental and spectral (IR, ^1^H NMR, mass) data. ^1^H NMR spectra of compounds **7** revealed in addition to the expected signals of the aromatic protons, and the protons of the two methyl groups, a singlet at δ 10.60–10.72 ppm, assigned to the –NH proton. The mass spectra of all products **5** exhibited, in each case, a molecular ion peak at the correct molecular weight for the respective compound (see Material and Methods).

Refluxing equimolar amounts of the appropriate arylazothiazole (**5**) and terephthalaldehyde (**6**) in EtOH containing catalytic amounts of glacial acetic acid gave the corresponding products, **3a**,**b**,**e**,**f**, which were identical in all respects (melting point (m.p.), mixed m.p. and IR spectra) with those obtained from reaction of compounds **1** with **2**.

**Scheme 3:**
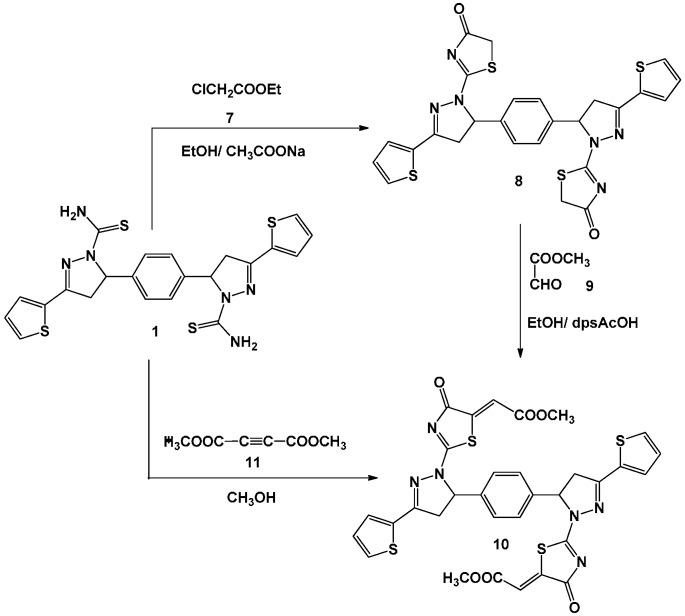
Synthesis of *bis*-thiazolone derivative **10**.

Finally, compound **1** reacted with ethyl chloroacetate (**7**) in ethanol in the presence of fused sodium acetate as a basic catalyst to give the *bis*-thiazolone derivative **8** (77% yield) (Scheme 3). Condensation of the latter product **8** with methyl glyoxalate (**9**) in absolute ethanol containing catalytic amounts of glacial acetic acid afforded dimethyl (2*Z*,2′*Z*)-2,2′-(2,2′-(5,5′-(1,4-phenylene)*bis*(3-(thiophen-2yl)-4,5-dihydro-1*H*-pyrazole-3,1-diyl))*bis*(4-oxothiazole-2(4*H*)-yl-5(4*H*)-ylidene))diacetate (**10**) as the end product in 66% yield. Compound **10** was also prepared by a one-step reaction of compound **1** with dimethyl acetylenedicarboxylate (DMAD) (**11**) in refluxing methanol as outlined in Scheme 3. The structure of compounds **10** was confirmed by elemental analyses and spectral data. For example, the ^1^H NMR spectrum of product **10** revealed the presence of a singlet at δ 6.68 ppm assigned to the olefinic CH proton of =CH–COOCH_3_ group, in addition to the signals of the aromatic, methyl, and ester protons. Its mass spectrum revealed a peak corresponding to its molecular ion at *m*/*z* 716 (see Material and Methods). The stereochemistry of the methylidene proton of compound **10** is *Z*-configuration according to literature reports [35,36,37].

### 2.2. Anti-Cancer Activity

The anticancer activity of some newly synthesized compounds was determined against a liver carcinoma cell line HepG2, using doxorubicin as a reference drug. Data generated were used to plot a dose-response curve of which the concentration (μM) of test compounds required to kill 50% of cell population (IC_50_) was determined. The cytotoxic activity was expressed as the mean IC_50_ of three independent experiments (Table 1), and the results revealed that all the tested compounds showed inhibitory activity to the tumor cell lines in a concentration dependent manner.

The results are represented in Table 1 showed the following:

1. The in vitro inhibitory activities of tested compounds against the human liver carcinoma (HepG2) have the descending order as follow: **3g** > **5e** > **3e** > **10** > **5f** > **3i** > **3f** > **3a** > **1** > **3a** > **3h** > **8** > **5b** > **3c** > **3b** > **3d**.
2. The thiazole derivatives **5a**,**b**,**e**,**f** have in vitro inhibitory activity greater than the *bis*-thiazole derivatives **3a**,**b**,**e**,**f** (**5a** > **3a**, **5b** > **3b**, **5e** > **3e**, and **5f** > **3f**).
3. The substituent at position 4 in the thiazole ring affect the in vitro inhibitory activity, while the thienyl ring has a greater effect than the methyl group, which has a greater effect than the phenyl group.
4. The introduction of electron-withdrawing group (nitro group > chlorine atom > bromine atom) at the fourth position of the phenyl group at position 5 in the thiazole ring enhances the antitumor activity. In contrast, the introduction of electron-donating group (methoxy group > methyl group) decreases the antitumor activity.

## 3. Materials and Methods

### 3.1. General Experimental Procedures

All melting points were determined on an electrothermal Gallenkamp apparatus (Bibby Sci. Lim. Stone, Staffordshire, UK). Solvents were distilled and dried by standard literature procedures prior to use. The IR spectra were measured on a Pye-Unicam SP300 instrument (Shimadzu, Tokyo, Japan) in potassium bromide discs. The ^1^H NMR and ^13^C NMR spectra were recorded on a Varian Mercury (Varian, Inc., Karlsruhe, Germany, 300 MHz for ^1^H NMR and 75 MHz for ^13^C NMR) and the chemical shifts were related to that of the solvent DMSO-*d*_6_. The mass spectra were recorded on GCMS-Q1000-EX Shimadzu (Tokyo, Japan), and the ionizing voltage was 70 eV. Elemental analyses were carried out by the Microanalytical Center of Cairo University, Giza, Egypt. Hydrazonoyl halides **2a**–**i** were prepared following a method from the literature [38].

### 3.2. Synthetic Procedures (See Supplementary Material Figures S1–S16)

#### 3.2.1. General Method for the Synthsis of 1,4-*Bis*(1-(4-substituted-5-((*E*)-aryldiazenyl)thiazol-2-yl)-3-(thiophen-2-yl)-4,5-dihydro-1*H*-pyrazol-5-yl)benzene **3a**–**i**

A mixture of *bis*-pyrazolylcarbothioamide **1** (0.336 g, 1 mmol) and the appropriate hydrazonoyl halides **2a**–**c** (2 mmol) in dioxane (20 mL) containing TEA (1 mL) was refluxed for 2–6 h (monitored by TLC) and allowed to cool, and the solid formed was filtered off, washed with EtOH, dried, and recrystallized from DMF to give **3a**–**i**. The products **3a**–**i** together with their physical constants is listed below.

*1,4-Bis(1-(4-methyl-5-((E)-phenyldiazenyl)thiazol-2-yl)-3-(thiophen-2-yl)-4,5-dihydro-1H-pyrazol-5-yl)-benzene* (**3a**). Orange solid; yield (71%); m.p. 192–194 °C (from DMF); IR (KBr) ν_max_: 3050, 2935 (CH), 1602 (C=N) cm^−1^; ^1^H NMR (DMSO-*d*_6_) δ: 2.59 (s, 6H, 2CH_3_), 3.15–3.19 (m, 2H, 2CH-pyrazoline), 3.77–3.82 (m, 2H, 2CH-pyrazoline), 5.92–5.96 (m, 2H, 2CH-pyrazoline), 7.02–7.82 (m, 16H, ArH and thiophene-H), 7.99 (s, 4H, ArH); ^13^C NMR (DMSO-*d*_6_) δ: 8.5 (CH_3_), 43.3 (CH_2_), 62.2 (CH), 121.2, 125.3, 125.8, 129.5, 130.4, 131.2, 131.7, 134.8, 136.2, 140.5, 141.8, 144.8 (Ar-C), 151.1, 158.3 (C=N); MS *m*/*z* (%): 780 (M^+^, 9), 341 (13), 185 (51), 119 (66), 78 (42), 51 (100). Anal. Calcd. For C_40_H_32_N_10_S_4_ (780.17): C, 61.51; H, 4.13; N, 17.93; Found: C, 61.39; H, 4.08; N, 17.72%.

*1,4-Bis(1-(4-methyl-5-((E)-p-tolyldiazenyl)thiazol-2-yl)-3-(thiophen-2-yl)-4,5-dihydro-1H-pyrazol-5-yl)benzene* (**3b**). Red solid; yield (74%); m.p. 180–182 °C (from DMF); IR (KBr) ν_max_: 3036, 2931 (CH), 1600 (C=N) cm^−1^; ^1^H NMR (DMSO-*d*_6_) δ: 2.35 (s, 6H, 2CH_3_), 2.57 (s, 6H, 2CH_3_), 3.04–3.09 (m, 2H, 2CH-pyrazoline), 3.56–3.60 (m, 2H, 2CH-pyrazoline), 5.92–5.94 (m, 2H, 2CH-pyrazoline), 7.15–7.86 (m, 14H, ArH and thiophene-H), 7.97 (s, 4H, ArH); ^13^C NMR (DMSO-*d*_6_) δ: 8.8, 20.4 (CH_3_), 45.3 (CH_2_), 67.2 (CH), 116.3, 125.6, 125.7, 129.0, 129.5, 129.6, 129.7, 131.6, 133.1, 133.4, 148.4, 149.9 (Ar-C), 160.5, 164.2 (C=N); MS *m*/*z* (%): 808 (M^+^, 5), 522 (11), 185 (44), 106 (23), 78 (80), 51 (100); Anal. Calcd. For C_42_H_36_N_10_S_4_ (808.20): C, 62.35; H, 4.48; N, 17.31; Found: C, 62.27; H, 4.41; N, 17.24%.

*1,4-Bis(1-(4-methyl-5-((E)-m-tolyldiazenyl)thiazol-2-yl)-3-(thiophen-2-yl)-4,5-dihydro-1H-pyrazol-5-yl)-benzene* (**3c**). Red solid; yield (68%); m.p. 164–166 °C (from DMF); IR (KBr) ν_max_: 3052, 2939 (CH), 1600 (C=N) cm^−1^; ^1^H NMR (DMSO-*d*_6_) δ: 2.32 (s, 6H, 2CH_3_), 2.62 (s, 6H, 2CH_3_), 3.03–3.07 (m, 2H, 2CH-pyrazoline), 3.53–3.57 (m, 2H, 2CH-pyrazoline), 5.90–5.94 (m, 2H, 2CH-pyrazoline), 7.06–7.82 (m, 14H, ArH and thiophene-H), 7.99 (s, 4H, ArH); ^13^C NMR (DMSO-*d*_6_) δ: 8.5, 20.0 (CH_3_), 45.6 (CH_2_), 66.3 (CH), 121.3, 125.7, 125.8, 127.7, 128.4, 128.6, 133.3, 138.5, 143.7, 144.5, 148.4, 148.8, 151.6 (Ar-C), 157.8, 163.6 (C=N); MS *m*/*z* (%): 808 (M^+^, 9), 631 (6), 512 (30), 464 (35), 185 (32), 92 (80), 87 (100), 51 (50); Anal. Calcd. For C_42_H_36_N_10_S_4_ (808.20): C, 62.35; H, 4.48; N, 17.31; Found C, 62.26; H, 4.40; N, 17.22%.

*1,4-Bis(1-(5-((E)-(4-methoxyphenyl)diazenyl)-4-methylthiazol-2-yl)-3-(thiophen-2-yl)-4,5-dihydro-1H-pyrazol-5-yl)benzene* (**3d**). Red solid; yield (70%); m.p. 190–192 °C (from DMF); IR (KBr) ν_max_: 3052, 2936 (CH), 1601 (C=N) cm^−1^; ^1^H NMR (DMSO-*d*_6_) δ: 2.59 (s, 6H, 2CH_3_), 3.04–3.07 (m, 2H, 2CH-pyrazoline), 3.79 (s, 6H, 2OCH_3_), 3.54–3.59 (m, 2H, 2CH-pyrazoline), 5.92–5.94 (m, 2H, 2CH-pyrazoline), 6.99–7.51 (m, 14H, ArH and thiophene-H), 7.90 (s, 4H, Ar-H); MS *m*/*z* (%): 840 (M^+^, 13), 631 (4), 348 (10), 220 (36), 185 (25), 109 (37), 78 (86), 51 (100); Anal. Calcd. For C_42_H_36_N_10_O_2_S_4_ (840.19): C, 59.98; H, 4.31; N, 16.65; Found C, 59.79; H, 4.26; N, 16.53%.

*1,4-Bis(1-(5-((E)-(4-chlorophenyl)diazenyl)-4-methylthiazol-2-yl)-3-(thiophen-2-yl)-4,5-dihydro-1H-pyrazol-5-yl)benzene* (**3e**). Red solid; yield (74%); m.p. 225–227 °C (from DMF); IR (KBr) ν_max_: 3052, 2948 (CH), 1602 (C=N) cm^−1^; ^1^H NMR (DMSO-*d*_6_) δ: 2.61 (s, 6H, 2CH_3_), 3.03–3.07 (m, 2H, 2CH-pyrazoline), 3.55–3.59 (m, 2H, 2CH-pyrazoline), 5.91–5.96 (m, 2H, 2CH-pyrazoline), 7.11–7.80 (m, 14H, ArH and thiophene-H), 7.98 (s, 4H, ArH); ^13^C NMR (DMSO-*d*_6_) δ: 8.4, 20.4 (CH_3_), 45.5 (CH_2_), 66.3 (CH), 115.5, 115.7, 125.2, 126.2, 126.3, 129.8, 131.9, 132.6, 135.2, 146.6, 155.7, 156.1 (ArC), 159.4, 162.6 (C=N); MS *m*/*z* (%): 850 (M^+^+2, 2), 848 (M^+^, 7), 631 (13), 404 (22), 243 (15), 185 (67), 117 (26), 78 (80), 51 (100); Anal. Calcd. For C_40_H_30_Cl_2_N_10_S_4_ (848.09): C, 56.53; H, 3.56; N, 16.48; Found: C, 56.44; H, 3.51; N, 16.36%.

*1,4-Bis(1-(5-((E)-(4-bromophenyl)diazenyl)-4-methylthiazol-2-yl)-3-(thiophen-2-yl)-4,5-dihydro-1H-pyrazol-5-yl)benzene* (**3f**). Red solid; yield (72%); m.p. 203–205 °C (from DMF); IR (KBr) ν_max_: 3050, 2933 (CH), 1601 (C=N) cm^−1^; ^1^H NMR (DMSO-*d*_6_) δ: 2.60 (s, 6H, 2CH_3_), 3.04–3.07 (m, 2H, 2CH-pyrazoline), 3.54–3.57 (m, 2H, 2CH-pyrazoline), 5.91–5.95 (m, 2H, 2CH-pyrazoline),7.30–7.86 (m, 14H, ArH and thiophene-H), 7.98 (s, 4H, ArH); ^13^C NMR (DMSO-*d*_6_) δ: 8.3 (CH_3_), 45.2 (CH_2_), 66.3 (CH), 116.1, 123.4, 124.2, 129.1, 130.9, 131.9, 132.2, 133.0, 141.3, 142.5, 142.8, 146.8, (Ar-C), 151.2, 162.1 (C=N); MS *m*/*z* (%): 937 (M^+^+2, 3), 935 (M^+^, 3), 657 (83), 592 (86), 490 (75), 414 (99), 185 (91), 78 (72), 51 (100); Anal. Calcd. For C_40_H_30_Br_2_N_10_S_4_ (935.99): C, 51.17; H, 3.22; N, 14.92; Found: C, 51.05; H, 3.13; N, 14.75%.

*1,4-Bis(1-(5-((E)-(4-nitrophenyl)diazenyl)-4-methylthiazol-2-yl)-3-(thiophen-2-yl)-4,5-dihydro-1H-pyrazol-5-yl)benzene* (**3g**). Red solid; yield (74%); m.p. 271–273 °C (from DMF); IR (KBr) ν_max_: 3056, 2033 (CH), 1602 (C=N) cm^−1^; ^1^H NMR (DMSO-*d*_6_) δ: 2.62 (s, 6H, 2CH_3_), 3.04–3.07 (m, 2H, 2CH-pyrazoline), 3.57–3.59 (m, 2H, 2CH-pyrazoline), 5.90–5.94 (m, 2H, 2CH-pyrazoline),7.08–7.79 (m, 14H, ArH and thiophene-H), 7.99 (s, 4H, ArH); ^13^C NMR (DMSO-*d*_6_) δ: 8.5 (CH_3_), 45.4 (CH_2_), 62.7 (CH), 114.8, 116.2, 123.5, 127.2, 128.6, 129.2, 139.4, 140.3, 141.0, 142.1, 142.8, 144.5, (ArC), 151.5, 162.1 (C=N); MS *m*/*z* (%): 870 (M^+^, 8), 657 (64), 530 (77), 442 (40), 334 (57), 185 (64), 78 (50), 51 (100); Anal. Calcd. For C_40_H_30_N_12_O_4_S_4_ (870.14): C, 55.16; H, 3.47; N, 19.30; Found: C, 55.07; H, 3.41; N, 19.21%.

*1,4-Bis(1-(4-phenyl-5-((E)-phenyldiazenyl)thiazol-2-yl)-3-(thiophen-2-yl)-4,5-dihydro-1H-pyrazol-5-yl)benzene* (**3h**). Orange solid; yield (77%); m.p. 260–262 °C (from DMF); IR (KBr) ν_max_: 3051, 2941 (CH), 1598 (C=N) cm^−1^; ^1^H NMR (DMSO-*d*_6_) δ: 3.09–3.10 (m, 2H, 2CH-pyrazoline), 3.57–3.59 (m, 2H, 2CH-pyrazoline), 5.93–5.96 (m, 2H, 2CH-pyrazoline), 7.47–7.94 (m, 26H, ArH and thiophene-H), 8.27 (s, 4H, ArH); MS *m*/*z* (%): 904 (M^+^, 3), 657 (64), 631 (7), 380 (12), 252 (37), 185 (42), 78 (77), 51 (100); Anal. Calcd. For C_50_H_36_N_10_S_4_ (904.20): C, 66.35; H, 4.01; N, 15.47; Found: C, 66.30; H, 4.13; N, 15.36%.

*1,4-Bis(1-(5-((E)-phenyldiazenyl)-4-(thiophen-2-yl)thiazol-2-yl)-3-(thiophen-2-yl)-4,5-dihydro-1H-pyrazol-5-yl)benzene* (**3i**). Orange solid; yield (76%); m.p. 248–250 °C (from DMF); IR (KBr) ν_max_: 3057, 2942 (CH), 1602 (C=N) cm^−1^; ^1^H NMR (DMSO-*d*_6_) δ: 3.05–3.08 (m, 2H, 2CH-pyrazoline), 3.55–3.58 (m, 2H, 2CH-pyrazoline), 5.92–5.95 (m, 2H, 2CH-pyrazoline), 7.05–7.94 (m, 22H, ArH and thiophene-H), 8.10 (s, 4H, ArH); ^13^C NMR (DMSO-*d*_6_) δ: 43.6 (CH_2_), 66.5 (CH), 119.0, 125.8, 126.0, 128.4, 128.5, 128.6, 128.9, 129.0, 129.4, 129.5, 130.8, 130.9, 131.1, 132.1, 132.3, 143.8 (ArC), 148.8, 162.8 (C=N); MS *m*/*z* (%): 916 (M^+^, 13), 522 (20), 409 (48), 285 (40), 111 (100), 78 (65), 51 (84); Anal. Calcd. For C_46_H_32_N_10_S_6_ (916.11): C, 60.24; H, 3.52; N, 15.27; Found: C, 60.15; H, 3.40; N, 15.19%.

#### 3.2.2. Synthesis of 4-Methyl-5-((*E*)-aryldiazenyl)-2-(2-(1-(thiophen-2-yl)ethylidene)hydrazinyl) Thiazole Derivatives **5a**,**b**,**e**,**f**

A mixture of thiosemicarbazone **4** (0.199 g, 1 mmol) and the appropriate hydrazonoyl halides **2** (1 mmol) in dioxane (20 mL) containing TEA (0.5 mL) was refluxed for 3–6 h and allowed to cool, and the solid formed was filtered off, washed with EtOH, dried, and recrystallized from DMF to give the corresponding arylazothiazoles **5a**,**b**,**e**,**f**. The products together with their physical constants are listed below.

*4-Methyl-5-((E)-phenyldiazenyl)-2-(2-(1-(thiophen-2-yl)ethylidene)hydrazinyl)thiazole* (**5a**). Red solid; yield (72%); m.p. 183–185 °C (from DMF); IR (KBr) ν_max_: 3427 (NH), 3043 (=C–H), 2931 (–C–H), 1603 (C=N) cm^−1^; ^1^H NMR (DMSO-*d*_6_) δ: 2.50 (s, 3H, CH_3_), 2.58 (s, 3H, CH_3_), 6.99–7.75 (m, 8H, ArH and thiophene-H), 10.67 (s, 1H, D_2_O-exchangeable, NH); ^13^C NMR (75 MHz, DMSO-*d*_6_) δ: 8.5, 15.5 (CH_3_), 114.2, 122.1, 128.0, 128.3, 129.2, 130.2, 130.8, 137.9, 143.4 (ArC), 160.4, 170.6, 177.6 (C=N); MS *m*/*z* (%): 341 (M^+^, 44), 238 (71), 106 (53), 78 (82), 51 (100); Anal. Calcd. for C_16_H_15_N_5_S_2_ (341.08): C, 56.28; H, 4.43; N, 20.51; Found: C, 56.22; H, 4.36; N, 20.40%.

*4-Methyl-2-(2-(1-(thiophen-2-yl)ethylidene)hydrazinyl)-5-((E)-p-tolyldiazenyl)thiazole* (**5b**). Red solid; yield (74%); m.p. 169–170 °C (from DMF); IR (KBr) ν_max_: 3427 (NH), 3042 (=C–H), 2928 (–C–H), 1602 (C=N) cm^−1^; ^1^H NMR (DMSO-*d*_6_) δ: 2.25 (s, 3H, CH_3_), 2.50 (s, 3H, CH_3_), 2.56 (s, 3H, CH_3_), 7.12–7.73 (m, 7H, ArH and thiophene-H), 10.60 (s, 1H, D_2_O-exchangeable, NH); ^13^C NMR (75 MHz, DMSO-*d*_6_) δ: 8.5, 15.5, 20.3 (CH_3_), 114.2, 128.0, 129.6, 130.0, 130.7, 131.1, 137.3, 141.1, 142.7 (ArC), 160.1, 177.3, 170.8 (C=N); MS *m*/*z* (%): 355 (M^+^, 20), 238 (53), 185 (77), 78 (69), 51 (100); Anal. Calcd. for C_17_H_17_N_5_S_2_ (355.09): C, 57.44; H, 4.82; N, 19.70; Found: C, 57.36; H, 4.77; N, 19.63%.

*5-((E)-(4-Chlorophenyl)diazenyl)-4-methyl-2-(2-(1-(thiophen-2-yl)ethylidene)hydrazinyl) thiazole* (**5e**). Red solid; yield (76%); m.p. 216–218 °C (from DMF); IR (KBr) ν_max_: 3427 (NH), 3043 (=C–H), 2931 (–C–H), 1603 (C=N) cm^−1^; ^1^H NMR (DMSO-*d*_6_) δ: 2.51 (s, 3H, CH_3_), 2.57 (s, 3H, CH_3_), 7.17–7.74 (m, 7H, ArH and thiophene-H), 10.71 (s, 1H, D_2_O-exchangeable, NH); ^13^C NMR (75 MHz, DMSO-*d*_6_) δ: 8.5, 15.4 (CH_3_), 115.6, 125.6, 128.0, 128.3, 129.1, 129.3, 138.7, 142.4, 142.5 (ArC), 160.6, 170.4, 177.6 (C=N); MS *m*/*z* (%): 377 (M^+^+2, 32), 375 (M^+^, 100), 222 (54), 186 (72), 78 (69), 51 (80); Anal. Calcd. for C_16_H_14_ClN_5_S_2_ (375.04): C, 51.12; H, 3.75; N, 18.63; Found: C, 51.03; H, 3.66; N, 18.51%.

*5-((E)-(4-Bromophenyl)diazenyl)-4-methyl-2-(2-(1-(thiophen-2-yl)ethylidene)hydrazinyl) thiazole* (**5f**). Red solid; yield (75%); m.p. 203–205 °C (from DMF); IR (KBr) ν_max_: 3441 (NH), 3043 (=C–H), 2937 (–C–H), 1601 (C=N) cm^−1^; ^1^H NMR (DMSO-*d*_6_) δ: 2.51 (s, 3H, CH_3_), 2.57 (s, 3H, CH_3_), 7.17–7.75 (m, 7H, ArH and thiophene-H), 10.72 (s, 1H, D_2_O-exchangeable, NH); ^13^C NMR (75 MHz, DMSO-*d*_6_) δ: 8.5, 15.5 (CH_3_), 116.1, 128.0, 130.3, 130.9, 131.9, 132.1, 138.8, 142.5, 142.8 (ArC), 160.6, 170.4, 177.6 (C=N); MS *m*/*z* (%): 421 (M^+^ + 2, 18), 419 (M^+^, 19), 302 (100), 185 (83), 78 (82), 51 (74); Anal. Calcd. for C_16_H_14_BrN_5_S_2_ (418.99): C, 45.72; H, 3.36; N, 16.66; Found: C, 45.55; H, 3.30; N, 16.42%.

#### 3.2.3. Alternate Synthesis of Compounds **3a**,**b**,**e**,**f**

A mixture of terephthalaldehyde (**6**) (0.134 g, 1 mmol) and the appropriate thiazole **5** (2 mmol) in EtOH (10 mL) containing 0.5 mL of glacial acetic acid was refluxed for 8 h and then cooled to room temperature. The solid precipitated was filtered off, washed with water, dried, and recrystallized from DMF to give the corresponding products, **3a**,**b**,**e**,**f**, which were identical in all respects (m.p., mixed m.p., and IR spectra) with those obtained from reaction of **1** with **2**.

#### 3.2.4. Synthesis of 2,2′-(5,5′-(1,4-phenylene)*bis*(3-(thiophen-2-yl)-4,5-dihydro-1*H*-pyrazole-3,1-diyl))*bis*(thiazol-4(5*H*)-one) (**8**)

A mixture of *bis*-pyrazolylcarbothioamide **1** (0.336 g, 1 mmol) and ethyl chloroacetate (**7**) (0.244 g, 2 mmol) in EtOH (20 mL) containing fused sodium acetate (1 g) was refluxed for 8 h and cooled to room temperature. The solid product was filtered off, washed with ethanol, and recrystallized from ethanol to afford the *bis*-thiazolone derivative **8** as yellow solid; yield (77%); m.p. 188–190 °C (from ethanol); IR (KBr) ν_max_: 3066, 2951 (CH), 1682 (C=O), 1600 (C=N) cm^−1^; ^1^H NMR (DMSO-*d*_6_) δ: 3.21–3.27 (m, 2H, 2CH-pyrazoline), 3.65–3.72 (m, 2H, 2CH-pyrazoline), 4.10 (s, 4H, 2CH_2_), 5.94–5.97 (m, 2H, 2CH-pyrazoline), 7.16–7.70 (m, 6H, thiophene-H), 7.97 (s, 4H, ArH); MS *m*/*z* (%): 576 (M^+^, 15), 445 (39), 283 (31), 185 (64), 78 (79), 51 (100); Anal. Calcd. for C_26_H_20_N_6_O_2_S_4_ (576.05): C, 54.15; H, 3.50; N, 14.57; Found: C, 54.15; H, 3.50; N, 14.57%.

#### 3.2.5. Synthesis of (2*Z*,2′*Z*)-dimethyl 2,2′-(2,2′-(5,5′-(1,4-phenylene)*bis*(5-(thiophen-2-yl)-4,5-dihydro-1*H*-pyrazole-3,1-diyl))*bis*(4-oxothiazole-2(4*H*)-yl-5(4*H*)-ylidene))diacetate (**10**)

A mixture of *bis*-thiazolone derivative **8** (0.580 g, 1 mmol), methyl glyoxalate (**9**) (0.176 g, 2 mmol), and glacial acetic acid (0.5 mL) in ethanol (20 mL) was refluxed for 6 h, left to cool, then poured gradually with stirring onto cold water. The solid formed was filtered off, washed with water, and crystallized from DMF to give compound **10** as canary yellow solid; yield (66%), m.p. 217–219 °C (from DMF); IR (KBr) ν_max_: 3061, 2936 (CH), 1742, 1682 (C=O), 1601 (C=N) cm^−1^; ^1^H NMR (DMSO-d_6_) δ: 3.54–3.57 (m, 2H, 2CH-pyrazoline), 3.71 (s, 6H, 2CH_3_), 3.84–3.87 (m, 2H, 2CH-pyrazoline), 5.97–6.01 (m, 2H, 2CH-pyrazoline), 6.68 (s, 2H, 2 =CHCOOCH_3_), 7.23–7.72 (m, 6H, thiophene-H), 7.99 (s, 4H, ArH); ^13^C NMR (DMSO-d_6_) δ: 44.0 (CH_2_), 52.4 (CH_3_), 63.9 (CH), 114.2, 116.3, 126.5, 128.4, 131.9, 133.5, 142.4, 146.5 (ArC), 158.0, 165.8, (C=N), 171.5, 177.1 (C=O); MS *m*/*z* (%): 716 (M^+^, 84), 631 (40), 314 (48), 185 (72), 116 (79), 51 (100). Anal. Calcd. For C_32_H_24_N_6_O_6_S_4_ (716.06): C, 53.62; H, 3.37; N, 11.72; Found: C, 53.53; H, 3.31; N, 11.59%.

#### 3.2.6. Alternate Synthesis of Compound **10**

To a solution of *bis*-pyrazolylcarbothioamide **1** (0.336 g, 1 mmol) in dry methanol (20 mL) was added dimethylacetylenedicarboxylate (**11**) (0.284 g, 2 mmol). The solution was refluxed for 4 h. The precipitated product after cooling was filtered, washed with methanol, and recrystallized from DMF to give product **10**, which was identical in all aspects (m.p., mixed m.p., and IR spectra) with that obtained from the reaction of **8** with **9**.

### 3.3. Anti-Tumor Activity

Human liver carcinoma (HepG2) cell lines were obtained from the American Type Culture Collection (ATCC, Rockville, MD). The cells were grown on RPMI-1640 medium supplemented with 10% inactivated fetal calf serum and 50 µg/mL of gentamicin. The cells were maintained at 37 °C in a humidified atmosphere with 5% CO_2_ and were sub-cultured two to three times a week.

For antitumor assays, the tumor cell lines were suspended in medium at concentration 5 × 10^4^ cell/well in corning^®^ 96-well plates (six replicates) to achieve eight concentrations for each compound. Six vehicle controls with media or 0.5% DMSO were run for each 96-well plate as a control. After incubating for 24 h, the numbers of viable cells were determined by the MTT test. Briefly, the media were removed from the 96-well plates and replaced with 100 µL of fresh culture RPMI 1640 medium without phenol red, followed by 10 µL of the 12 Mm MTT stock solutions (5 mg of MTT in 1 mL of PBS) to each well, including the untreated controls. The 96-well plates were then incubated at 37 °C and 5% CO_2_ for 4 h. An 85-µL aliquot of the media was removed from the wells, and 50 µL of DMSO was added to each well and mixed thoroughly with the pipette and incubated at 37 °C for 10 min. Then, the optical density was measured at 590 nm with the microplate reader (SunRise, TECAN, Inc, Männedorf, Switzerland) to determine the number of viable cells and the percentage of viability was calculated as (1 − (ODt/ODc)) × 100%, where ODt is the mean optical density of untreated cells. The relation between surviving cells and drug concentration is plotted to obtain the survival curve of each tumor cell line after treatment with the specified compound. The 50% inhibitory concentration (IC_50_), the concentration required to cause toxic effects in 50% of intact cells, was estimated from graphic plots of the dose-response curve for each concentration using Graphed Prism software (San Diego, CA, USA) [39,40,41].

## 4. Conclusions

In our present work, a new series of 2-ethylidenehydrazono-5-arylazothiazoles and 2-ethylidene-hydrazono-5-arylazothiazolones were synthesized from a reaction of ethylidene-thiosemicarbazide with various hydrazonoyl halides. The structures of the newly synthesized compounds were established on the basis of spectroscopic evidences and their synthesis by alternative methods. The in-vitro growth inhibitory activity of the synthesized compounds against hepatocellular carcinoma (HepG2) cell lines was investigated in comparison with doxorubicin as a standard drug using an MTT assay, and the results revealed promising activities of compounds **3g**, **5e**, **3e**, **10**, **5f**, **3i**, and **3f** with an IC_50_ equal to 1.37 ± 0.15, 1.41 ± 0.17, 1.62 ± 0.20, 1.86 ± 0.20, 1.93 ± 0.08, 2.03 ± 0.25, and 2.09 ± 0.19 μM, respectively.

## Figures and Tables

**Table 1:**
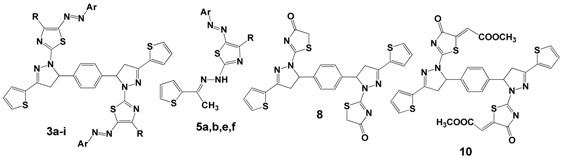
The in vitro inhibitory activity of tested compounds against a liver carcinoma cell line HepG2 expressed as IC50 values (μM) ± standard deviation from six replicates.

Compound No.	R	Ar	IC_50_ (μM)
**1**	-	-	4.05 ± 0.17
**3a**	Me	C_6_H_5_	6.29 ± 0.22
**3b**	Me	4-MeC_6_H_4_	18.2 ± 0.14
**3c**	Me	3-MeC_6_H_4_	17.6 ± 0.09
**3d**	Me	4-MeOC_6_H_4_	52.4 ± 0.17
**3e**	Me	4-ClC_6_H_4_	1.62 ± 0.20
**3f**	Me	4-BrC_6_H_4_	2.09 ± 0.19
**3g**	Me	4-NO_2_C_6_H_4_	1.37 ± 0.15
**3h**	C_6_H_5_	C_6_H_5_	10.49 ± 0.22
**3i**	C_4_H_4_S	C_6_H_5_	2.03 ± 0.25
**5a**	Me	C_6_H_5_	5.13 ± 0.09
**5b**	Me	4-MeC_6_H_4_	14.29 ± 0.22
**5e**	Me	4-ClC_6_H_4_	1.41 ± 0.17
**5f**	Me	4-BrC_6_H_4_	1.93 ± 0.08
**8**	-	-	12.37 ± 0.14
**10**	-	-	1.86 ± 0.20
**Doxorubicin**	-	-	0.72 ± 0.18

## Supplementary Materials

Supplementary materials can be found www.mdpi.com/1422-0067/17/9/1499/s1.

## References

[1] Kulandasamy R., Adhikari A.V., Stables J.P. (2009). A new class of anticonvulsants possessing 6 Hz activity: 3,4-Dialkyloxy thiophene *bis*-hydrazones. Eur. J. Med. Chem..

[2] Asiri A.M., Khan S.A. (2011). Synthesis and anti-bacterial activities of a *bis*-chalcone derived from thiophene and its *bis*-cyclized products. Molecules.

[3] Murakami N., Takase H., Saito T., Iwata K., Miura H., Naruse T. (1998). Effects of a novel non-steroidal anti-inflammatory drug (M-5011) on bone metabolism in rats with collagen induced arthritis. Eur. J. Pharmacol..

[4] Lu X., Wan B., Franzblau S.G., You Q. (2011). Design, synthesis and anti-tubercular evaluation of new 2-acylated and 2-alkylated amino-5-(4-(benzyloxy)phenyl)thiophene-3-carboxylic acid derivatives. Part 1. Eur. J. Med. Chem..

[5] Corral C., Lissavetzky J., Manzanares I., Darias V., Expósito-Orta M.A., Conde J.A.M., Sánchez-Mateo C.C. (1999). Synthesis and preliminary pharmacological evaluation of thiophene analogues of viloxazine as potential antidepressant drugs. Bioorg. Med. Chem..

[6] Ishikawa F., Kosasayama A., Yamaguchi H., Watanabe Y., Saegusa J., Shibamura S., Sakurna K., Ashida S., Abiko Y. (1981). Cyclic guanidines. 14. Imidazo[1,2-*a*]thienopyrimidin-2-one derivatives as blood platelet aggregation inhibitors. J. Med. Chem..

[7] Ozdemir A., Turan-Zitouni G., Kaplancıklı Z.A., Revial G., Guven K. (2007). Synthesis and antimicrobial activity of 1-(4-aryl-2-thiazolyl)-3-(2-thienyl)-5-aryl-2-pyrazoline derivatives. Eur. J. Med. Chem..

[8] Turan-Zitouni G., Ozdemir A., Guven K. (2005). Synthesis of some 1[(*N*,*N*-disubstitutedthio-carbamoylthio)acetyl]-3-(2-thienyl)-5-aryl-2-prazoline derivatives and investigation of their antibacterial and antifungal activities. Arch. Pharm..

[9] Flora F., Hosni H.H., Girgis A.S. (2006). Novel *bis*(1-acyl-2-pyrazolines)of potential anti-inflammatory and molluscicidal properties. Bioorg. Med. Chem..

[10] Gomha S.M., Riyadh S.M., Abdalla M.M. (2015). Solvent-drop grinding method: Efficient synthesis, DPPH radical scavenging and anti-diabetic activities of chalcones, *bis*-chalcones, azolines, and *bis*-azolines. Curr. Org. Synth..

[11] Kreutzberger A., Kolter K. (1986). Antiviral agents. XXVII: Aminomethynylation of 5-oxo-2-pyrazoline-3-carboxylic acid derivatives. Arch. Pharm..

[12] Shaharyar M., Siddiqui A.A., Ali M.M., Sriram D., Yogeeswari P. (2006). Synthesis and in vitro antimycobacterial activity of *N*^1^-nicotinoyl-3-(-4′-hydroxy-3′-methyl phenyl)-5-[(sub)phenyl]-2-pyrazolines. Bioorg. Med. Chem. Lett..

[13] Abid M., Azam A. (2006). Synthesis, characterization and antimoebic activity of 1-(thiazolo[4,5-*b*]-quinoxalin-2-yl)-3-phenyl-2-pyrazoline derivatives. Bioorg. Med. Chem. Lett..

[14] Abid M., Azam A. (2005). 1-*N*-substituted thiocarbamoyl-3-phenyl-2-pyrazolines: Synthesis and in vitro antiamoebic activities. Eur. J. Med. Chem..

[15] Ozdemir Z., Kandilci H.B., Gumusel B., Calis U., Bilgin A.A. (2007). Synthesis and studies on antidepressant and anticonvulsant activities of some 3-(2-furyl)-pyrazoline derivatives. Eur. J. Med. Chem..

[16] Amir M., Kumar H., Khan S.A. (2008). Synthesis and pharmacological evaluation of pyrazoline derivatives as new anti-inflammatory and analgesic agents. Bioorg. Med. Chem. Lett..

[17] Karegoudar P., Karthikeyan M.S., Prasad D.J., Mahalinga M., Holla B.S., Kumari N.S. (2008). Synthesis of some novel 2,4-disubstituted thiazoles as possible antimicrobial agents. Eur. J. Med. Chem..

[18] Shih M.H., Ying K.F. (2004). Syntheses and evaluation of antioxidant activity of sydnonyl substituted thiazolidinone and thiazoline derivatives. Bioorg. Med. Chem..

[19] Shiradkar M., Kumar G.V.S., Dasari V., Tatikonda S., Akula K.C., Shah R. (2007). Clubbed triazoles A novel approach to antitubercular drugs. Eur. J. Med. Chem..

[20] Amin K.M., Rahman A.D.E., Al-Eryani Y.A. (2008). Synthesis and preliminary evaluation of some substituted coumarins as anticonvulsant agents. Bioorg. Med. Chem..

[21] Gomha S.M., Riyadh S.M., Abbas I.M., Bauomi M.A. (2013). Synthetic utility of ethylidene-thiosemicarbazide: Synthesis and and anti-cancer activity of 1,3-thiazines and thiazoles with imidazole moiety. Heterocycles.

[22] Gomha S.M., Salah T.A., Hassaneen H.M.E., Abdel-aziz H., Khedr M.A. (2016). Synthesis, characterization and molecular docking of novel bioactive thiazolyl-thiazole derivatives as promising cytotoxic antitumor drug. Molecules.

[23] Gomha S.M., Riyadh S.M., Mahmmoud E.A., Elaasser M.M. (2015). Chitosan-grafted-poly(4-vinylpyridine) as a novel copolymer basic catalyst for synthesis of arylazothiazoles and 1,3,4-thiadiazoles under microwave irradiation. Chem. Heterocycl. Comp..

[24] Gomha S.M., Khalil K.D. (2012). A convenient ultrasound-promoted synthesis and cytotoxic activity of some new thiazole derivatives bearing a coumarin nucleus. Molecules.

[25] Gomha S.M., Salah T.A., Abdelhamid A.O. (2015). Synthesis, characterization and pharmacological evaluation of some novel thiadiazoles and thiazoles incorporating pyrazole moiety as potent anticancer agents. Monatsh. Chem..

[26] Gomha S.M., Badrey M.G., Edrees M.M. (2016). Heterocyclization of a *bis*-thiosemicarbazone of 2,5-diacetyl-3,4-disubstituted-thieno[2,3-*b*]thiophenebis-thiosemicarbazones leading to *bis*-thiazoles and *bis*-1,3,4-thiadiazoles as anti-breast cancer agents. J. Chem. Res..

[27] Gomha S.M., Zaki Y.H., Abdelhamid A.O. (2015). Utility of 3-acetyl-6-bromo-2*H*-chromen-2-one for synthesis of new heterocycles as potential anticancer agents. Molecules.

[28] Dawood K.M., Gawad H.A., Rageb E.A., Ellithey M., Mohamed H.A. (2006). Synthesis, anticonvulsant, and anti-inflammatory evaluation of some new benzotriazole and benzofuran-based heterocycles. Bioorg. Med. Chem..

[29] Altalbawy F.M.A. (2013). Synthesis and antimicrobial evaluation of some novel *bis*-α,β-unsaturated ketones, nicotinonitrile, 1,2-dihydropyridine-3-carbonitrile, fused thieno[2,3-*b*]pyridine and pyrazolo-[3,4-*b*] pyridine derivatives. Int. J. Mol. Sci..

[30] Kheder N.A., Altalbawy F.M.A. (2016). Synthesis, in vitro antimicrobial, anticancer evaluation of some novel *bis*-cyanoacrylamide and *bis*-azoles derivatives. Int. J. Pharm. Pharm. Sci..

[31] Altalbawy F.M.A. (2015). Synthesis, in vitro antimicrobial, anticancer evaluation of some new pyridazines and polyfunctionally substituted heterocyclic compounds. Asian J. Chem..

[32] Altıntop M.D., Mohsen U., Karaca A., Canturk Z., Ozdemir A. (2014). Synthesis and Evaluation of *bis*-pyrazoline Derivatives as Potential Antimicrobial Agents. Lett. Drug Des. Discov..

[33] Abdelhamid A.O., Sayed A.R. (2007). Reaction of hydrazonoyl halides 52: Synthesis and antimicrobial activity of some new pyrazolines and 1,3,4-thiadiazolines. Phosphorus Sulfur Silicon.

[34] Lima G.M., Neto J.L., Beraldo H., Siebald H.G.L., Duncalf D.J. (2002). Structural and spectral studies of thiosemicarbazones derived from 2-acetylthiophene. J. Mol. Struct..

[35] Benmohammed A., Khoumeri O., Djafri A., Terme T., Vanelle P. (2014). Synthesis of novel highly functionalized 4-thiazolidinone derivatives from 4-phenyl-3-thiosemicarbazones. Molecules.

[36] Aly A.A., Brown A.B., Ramadan M., Gamal-Eldeen A.M., Abdel-Aziz M., El-Din G., Abuo-Rahma A.A., Radwan M.F. (2010). Thieno[2,3-*d*]pyrimidines in the Synthesis of antitumor and antioxidant agents. Arch. Pharm. Chem. Life Sci..

[37] Gomha S.M., Badrey M.G. (2013). A convenient synthesis of some new thiazole and pyrimidine derivatives incorporating a naphthalene moiety. J. Chem. Res..

[38] Shawali A.S., Gomha S.M. (2000). A new entry for short and regioselective synthesis of [1,2,4]triazolo-[4,3-*b*][1,2,4]-triazin-7(1*H*)-ones. Adv. Synth. Catal..

[39] Mosmann T. (1983). Rapid colorimetric assay for cellular growth and survival: Application to proliferation and cytotoxicity assays. J. Immunol. Methods.

[40] Gomha S.M., Riyadh S.M., Mahmmoud E.A., Elasser M.M. (2015). Synthesis and anticancer activities of thiazoles, 1,3-thiazines, and thiazolidine using chitosan-grafted-poly(vinylpyridine) as basic catalyst. Heterocycles.

[41] Srivastava S.K., Jha A., Agarwal S.K., Mukherjee R., Burman A.C. (2007). Synthesis and structure-activity relationships of potent antitumor active quinoline and naphthyridine derivatives. Anti-Cancer Agents Med. Chem..

