# Supplementary material for: Synthesis and Characterization of Some New Bis-Pyrazolyl-Thiazoles Incorporating the Thiophene Moiety as Potent Anti-Tumor Agents
Source: Int J Mol Sci. 2016 Sep 7;17(9):1499. doi: 10.3390/ijms17091499 (PMC5037776; doi:10.3390/ijms17091499)
Supplement: Supplementary file 1 [file ijms-17-01499-s001.pdf]

Supplementary Materials: Synthesis and Characterization of Some New *Bis*-Pyrazolyl-Thiazoles Incorporating the Thiophene Moiety as Potent Anti-Tumor Agents

Sobhi M. Gomha, Mastoura M. Edrees and Farag M. A. Altalbawy

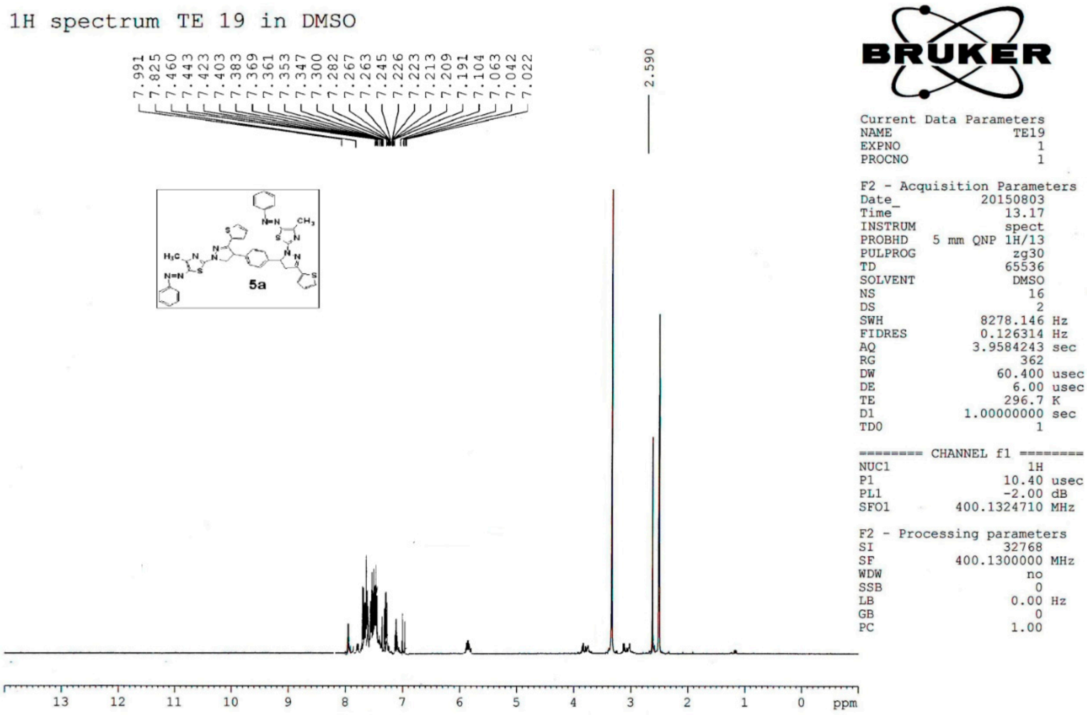

Figure S1. ^{1}H NMR spectrum of compound **3a**.

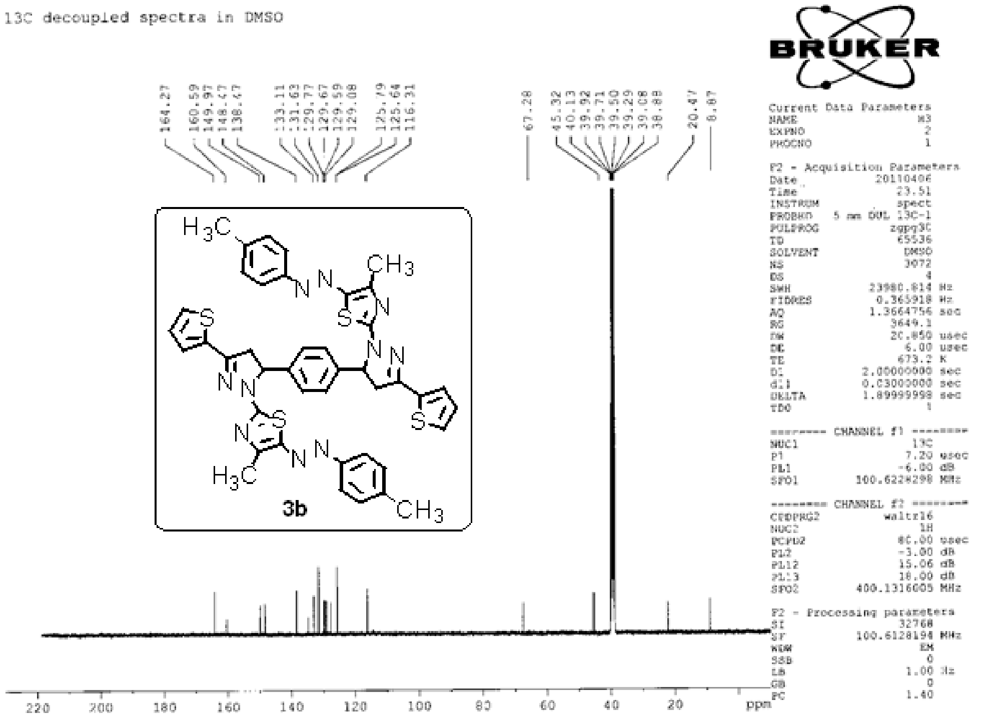

Figure S2. ^{13}C NMR spectrum of compound **3b**.

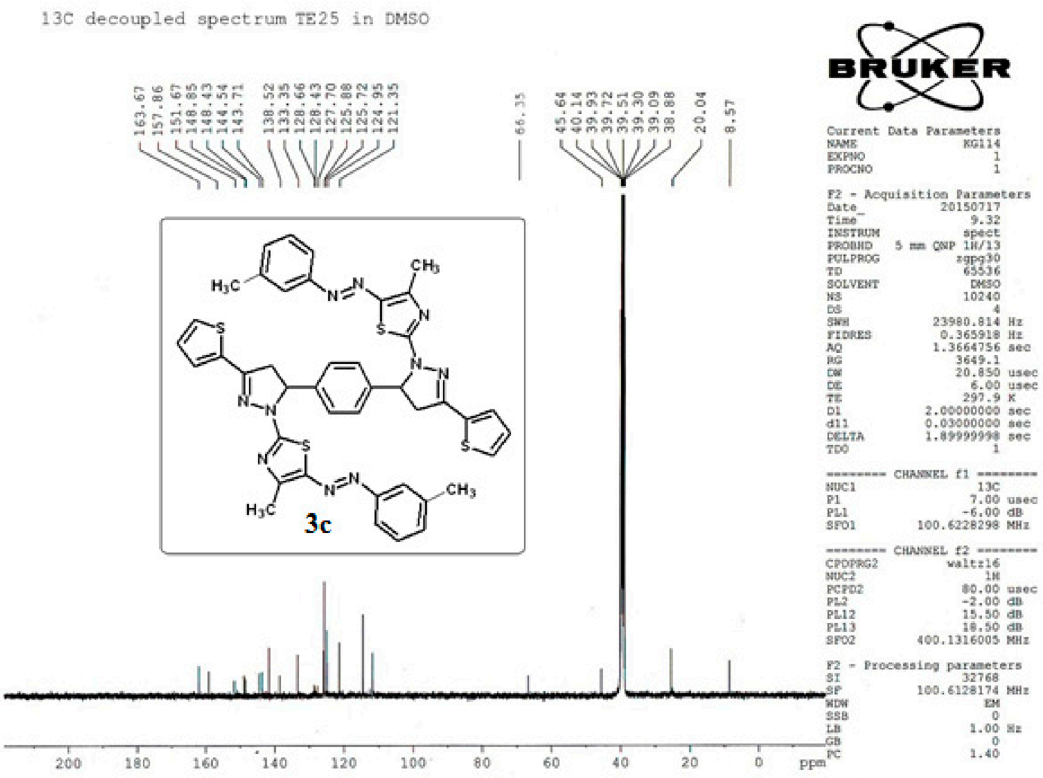

Figure S3. ^{13}C NMR spectrum of compound **3c**.

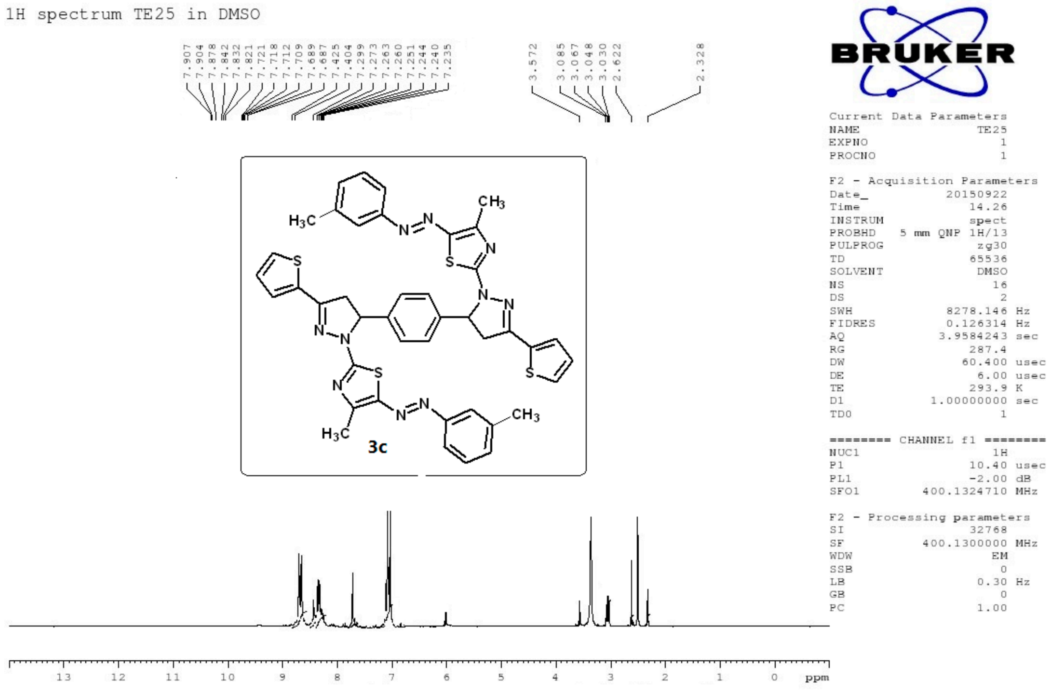

Figure S4. ^{1}H NMR spectrum of compound **3c**.

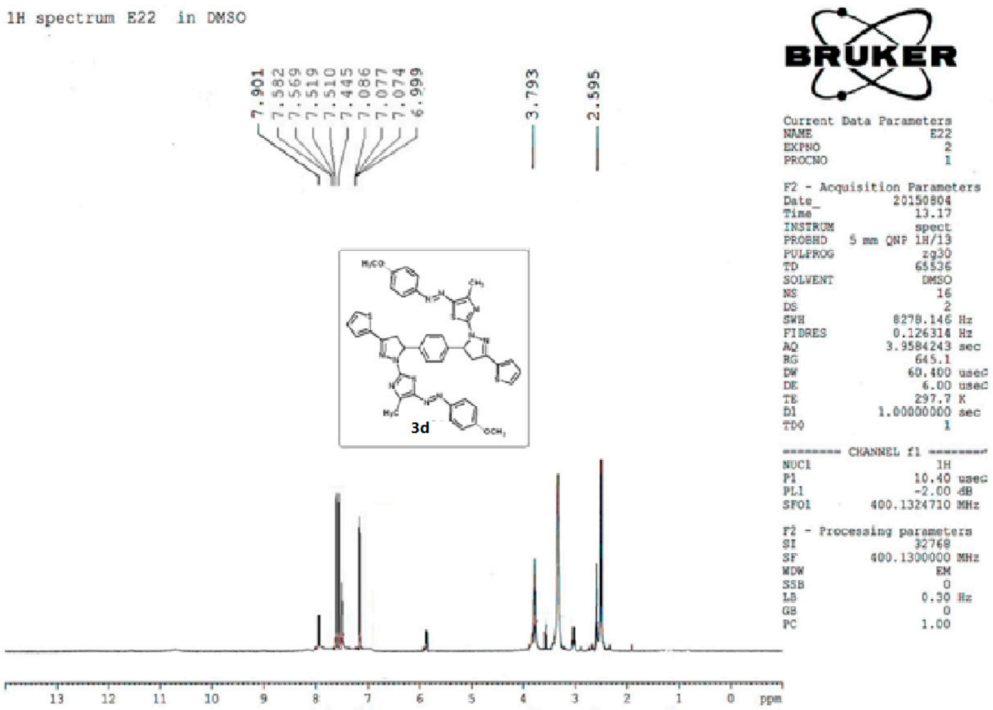

Figure S5. ^{1}H NMR spectrum of compound **3d**.

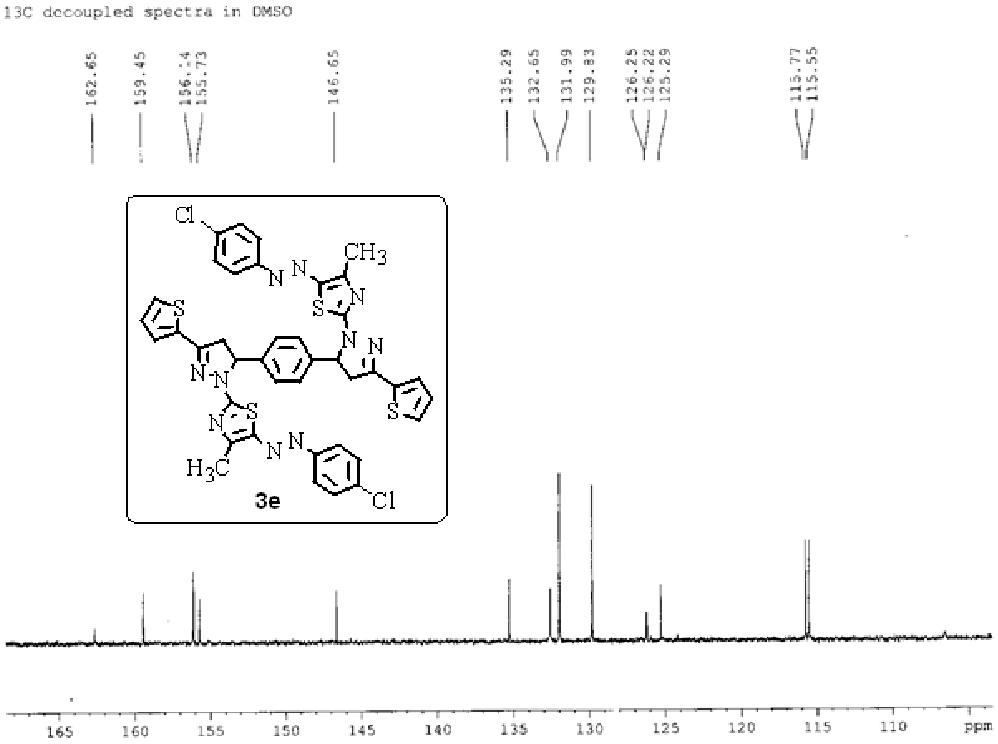

Figure S6. ^{13}C NMR spectrum of compound **3e**.

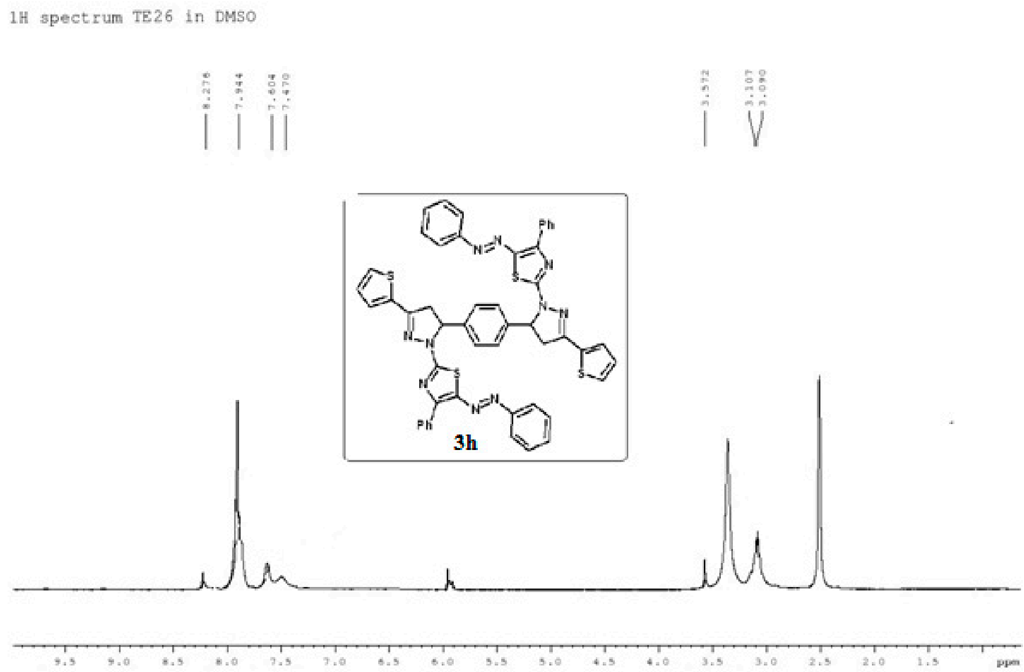

Figure S7. ^{1}H NMR spectrum of compound **3h**.

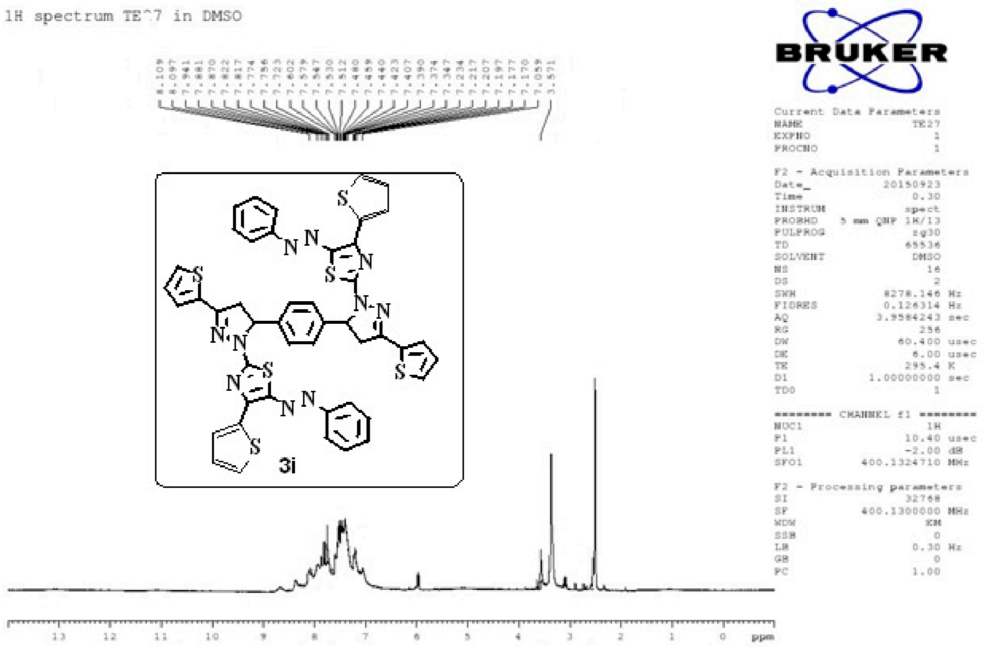

Figure S8. ^{1}H NMR spectrum of compound **3i**.

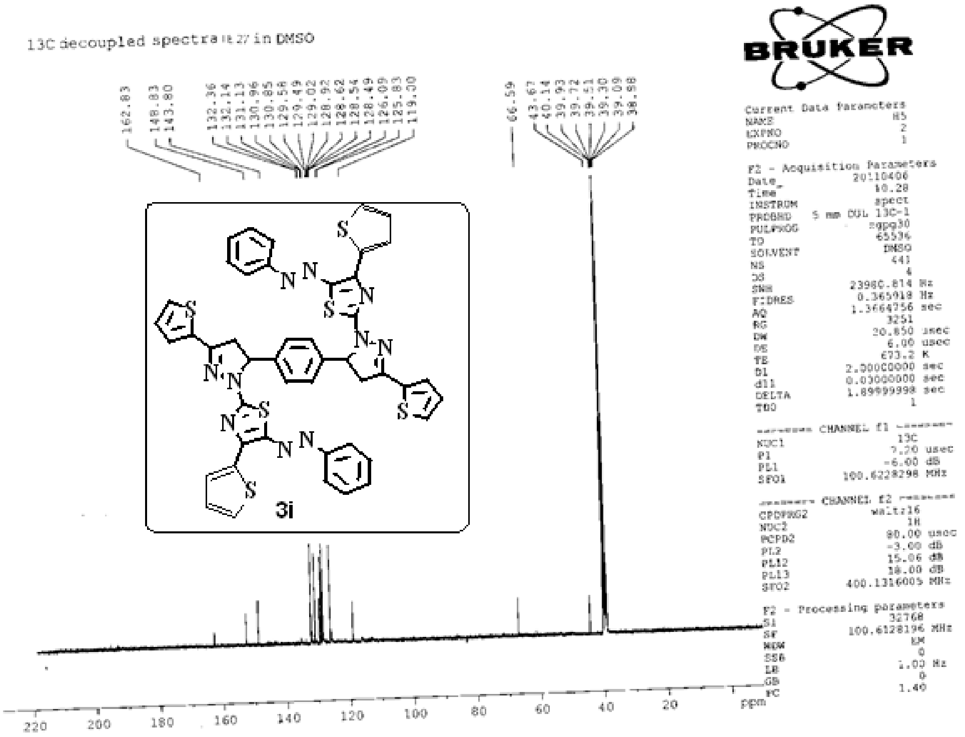

Figure S9. ^{13}C NMR spectrum of compound **3i**.

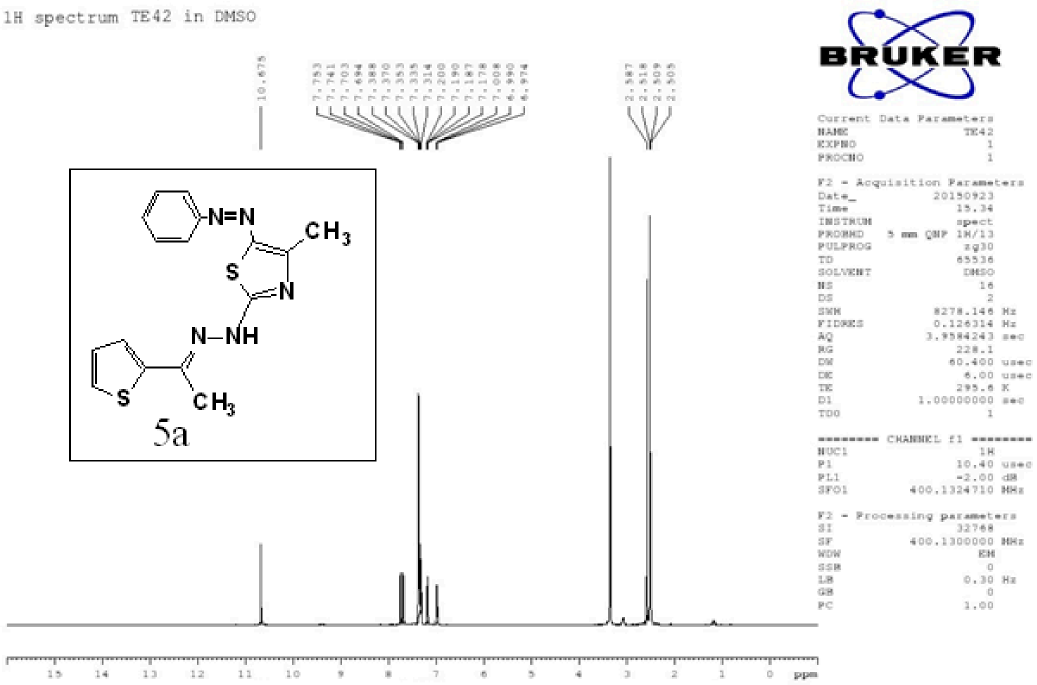

Figure S10. ^{1}H NMR spectrum of compound **5a**.

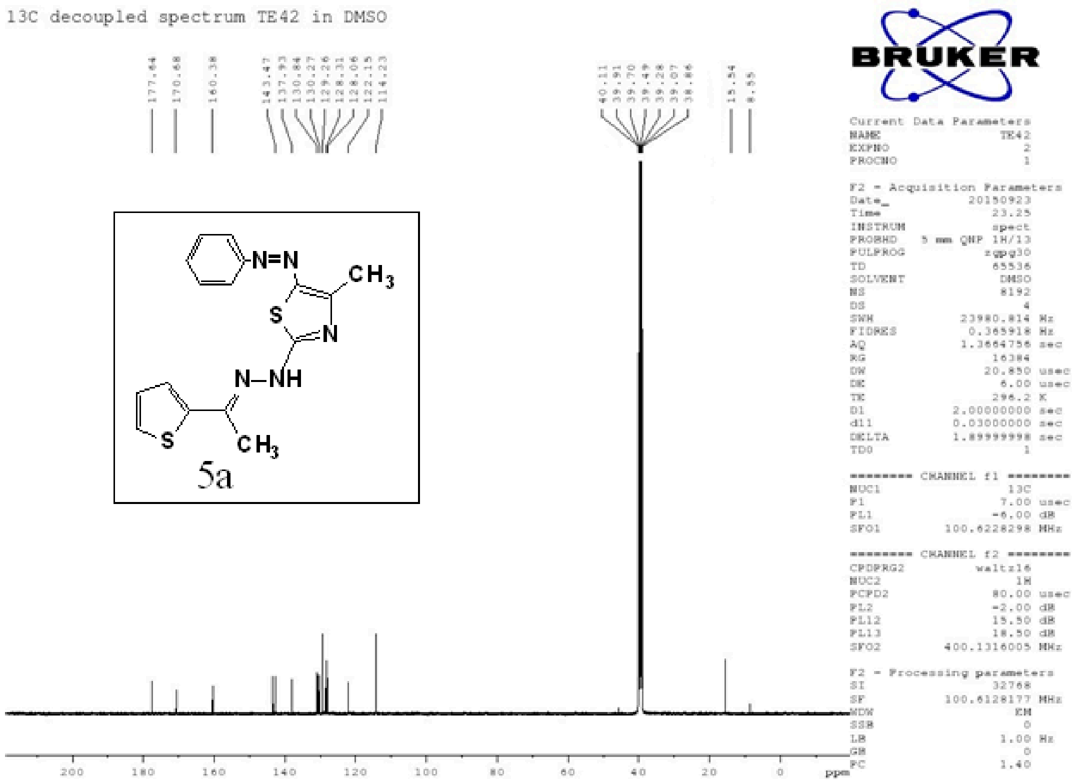

Figure S11. ^{13}C NMR spectrum of compound **5a**.

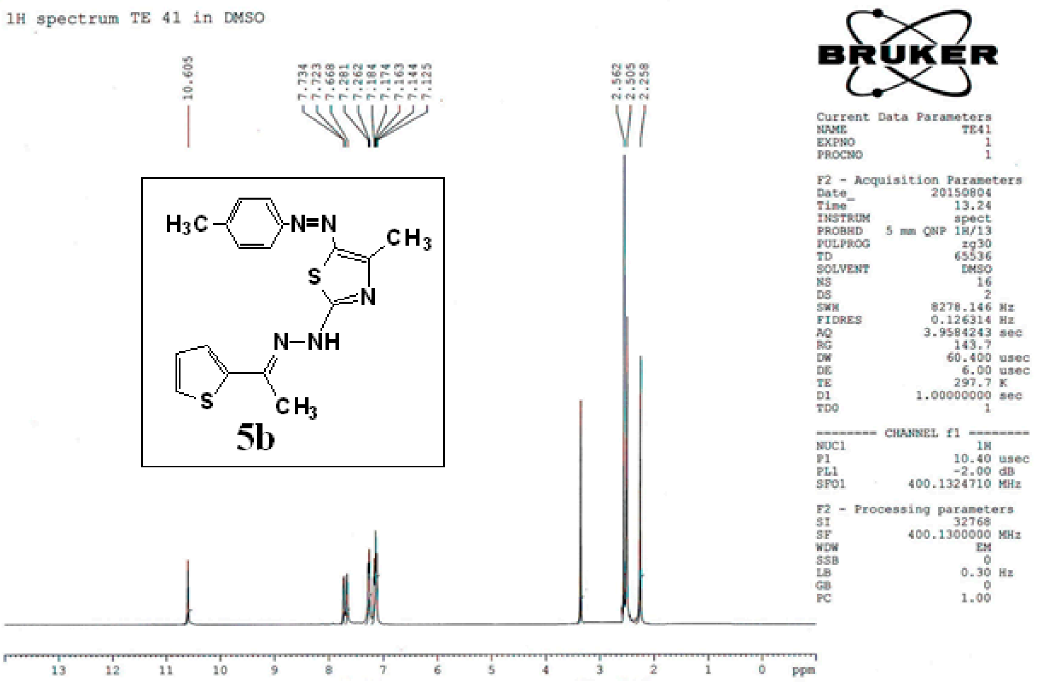

Figure S12. ^{1}H NMR spectrum of compound **5b**.

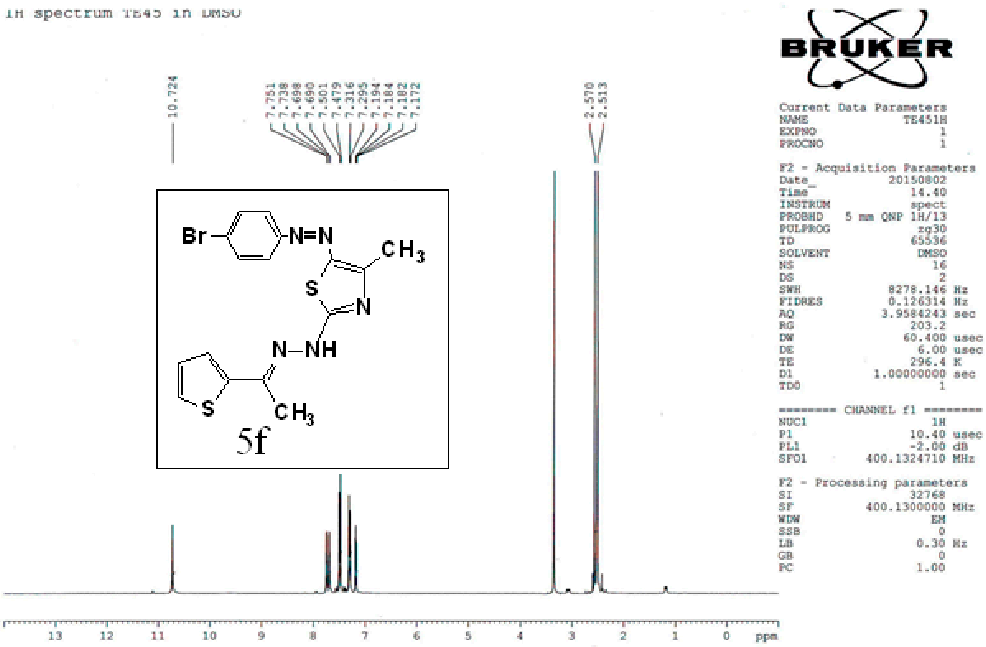

Figure S13. ^{1}H NMR spectrum of compound **5f**.

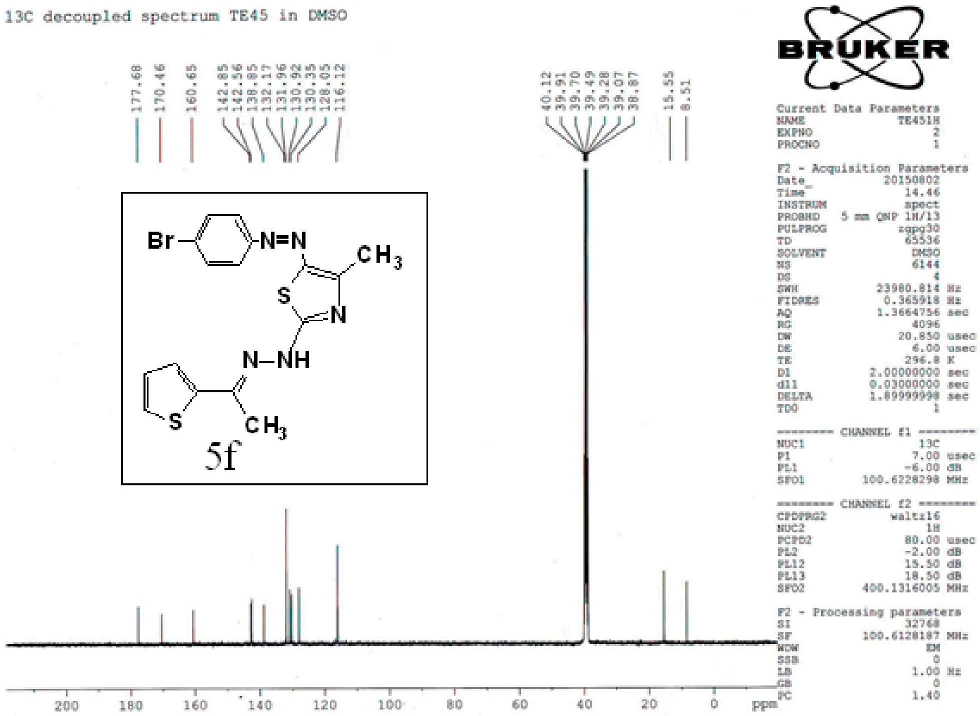

Figure S14. ^{13}C NMR spectrum of compound **5f**.

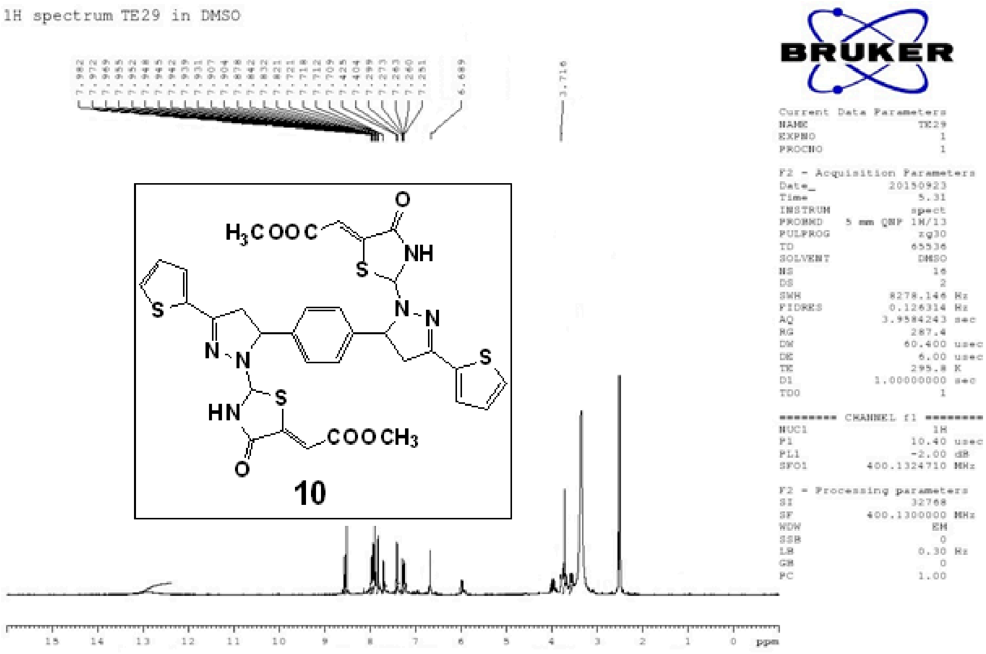

Figure S15. ^{1}H NMR spectrum of compound **10**.

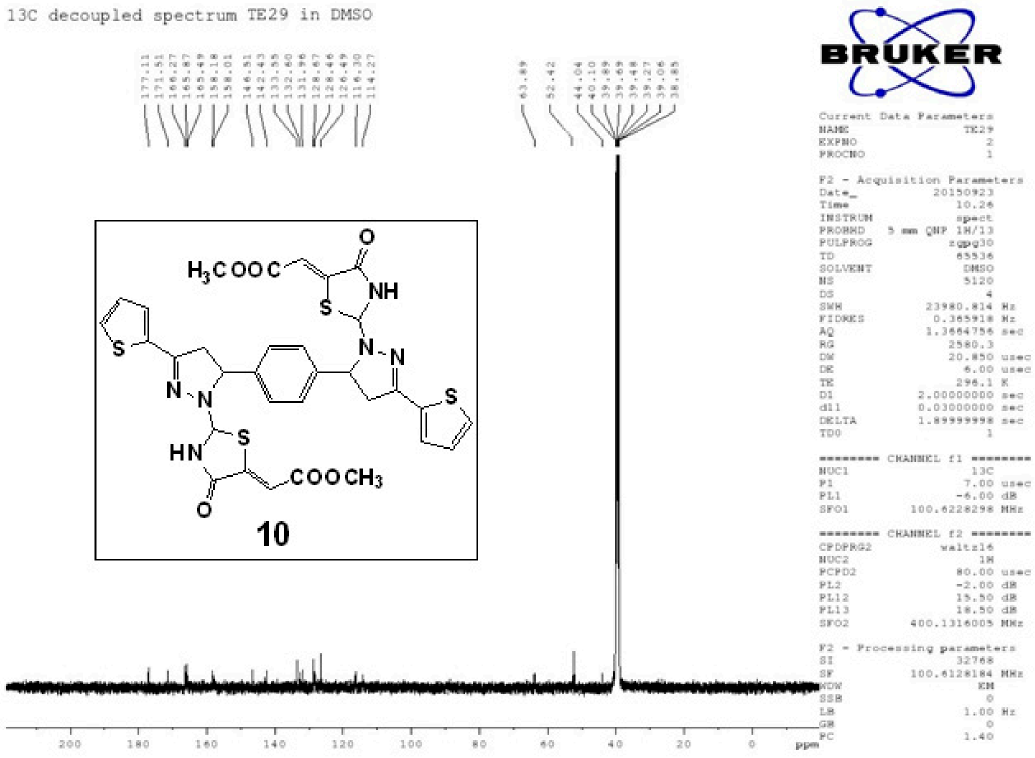

Figure S16. ^{13}C NMR spectrum of compound **10**.
